# Astaxanthin as a Potent Antioxidant for Promoting Bone Health: An Up-to-Date Review

**DOI:** 10.3390/antiox12071480

**Published:** 2023-07-24

**Authors:** Iswari Davan, Sharida Fakurazi, Ekram Alias, Nurul ‘Izzah Ibrahim, Ng Min Hwei, Haniza Hassan

**Affiliations:** 1 Department of Human Anatomy, Faculty of Medicine and Health Sciences, Universiti Putra Malaysia (UPM), Serdang 43400, Malaysia; gs64124@student.upm.edu.my (I.D.); sharida@upm.edu.my (S.F.); 2 Department of Biochemistry, Faculty of Medicine, Universiti Kebangsaan Malaysia (UKM), Jalan Yaacob Latiff, Bandar Tun Razak, Kuala Lumpur 56000, Malaysia; ekram.alias@ppukm.ukm.edu.my; 3 Department of Pharmacology, Faculty of Medicine, Universiti Kebangsaan Malaysia (UKM), Jalan Yaacob Latiff, Bandar Tun Razak, Kuala Lumpur 56000, Malaysia; 4 Centre for Tissue Engineering and Regenerative Medicine, Universiti Kebangsaan Malaysia (UKM), Jalan Yaacob Latiff, Bandar Tun Razak, Kuala Lumpur 56000, Malaysia; angela@ppukm.ukm.edu.my

**Keywords:** astaxanthin, antioxidant, bone, osteoporosis, osteoarthritis, osteosarcoma

## Abstract

In recent years, bone loss and its associated diseases have become a significant public health concern due to increased disability, morbidity, and mortality. Oxidative stress and bone loss are correlated, where oxidative stress suppresses osteoblast activity, resulting in compromised homeostasis between bone formation and resorption. This event causes upregulation of bone remodeling turnover rate with an increased risk of fractures and bone loss. Therefore, supplementation of antioxidants can be proposed to reduce oxidative stress, facilitate the bone remodeling process, suppress the initiation of bone diseases, and improve bone health. Astaxanthin (3,3′-dihydroxy-4-4′-diketo-β-β carotene), a potent antioxidant belonging to the xanthophylls family, is a potential ROS scavenger and could be a promising therapeutic nutraceutical possessing various pharmacological properties. In bone, astaxanthin enhances osteoblast differentiation, osteocytes numbers, and/or differentiation, inhibits osteoclast differentiation, cartilage degradation markers, and increases bone mineral density, expression of osteogenic markers, while reducing bone loss. In this review, we presented the up-to-date findings of the potential anabolic effects of astaxanthin on bone health in vitro, animal, and human studies by providing comprehensive evidence for its future clinical application, especially in treating bone diseases.

## 1. Introduction

Bone diseases are classified as a major global health issue along with other non-communicable diseases, including cardiovascular diseases, diabetes, and cancers. These diseases have been on the rise in recent times and account for 200 million people being affected worldwide [1]. The prevalence of bone diseases can be attributed to reduced bone mineral density, microstructure deterioration, and dysregulation in remodeling of bone, which increases the risk of bone fracture. This has a devastating impact on the overall physical and mental health of an individual and is usually associated with high rates of morbidity and functional disability [2].

Bone health is an important aspect in every individual as it has various functions, such as locomotion, support, protection of various organs, and serves as a reservoir of minerals, particularly calcium and phosphate [3]. Bone is considered a complex and dense tissue of organic and inorganic components. The organic component comprises collagenous proteins, predominated by type I collagen and non-collagenous proteins, while the inorganic part consists of calcium and phosphorus minerals in the form of crystalline hydroxyapatite: [Ca_3_(PO_4_)_2_]3Ca (OH)_2_ and water [4,5].

Based on the structure, bone could be categorized into two types: Cancellous (trabecular) and cortical bone (compact). Trabecular bone has a structure like trabeculae and accounts for about 20% of the volume of the human skeleton. It is demonstrated as highly porous tissue, indicating low density and propensity to fracture [6]. Cortical bone consists of a primary lamellar structure, which forms a secondary structure due to the remodeling process. It is the denser tissue usually found on the surface of bones and composed of arranged osteons, the cylindrical features containing a central longitudinal cavity called the Haversian canal, and usually have low porosity <5%, which then translates to a high density [7]. This Haversian canal supplies nutrients and oxygen through primary blood flow to osteocytes and other bone cells [8]. Bone constantly undergoes renewal through the remodeling process responsible for maintaining the strength and shape of a bone.

This remodeling process requires a balance action of three types of bone cells, the bone-lining cells, osteoblasts, osteoclasts, and osteocytes, which make up the anatomical structure called basic multicellular unit (BMU). Bone remodeling is crucial as it is a physiological process of removing mineralized bone (resorption) by osteoclasts and the formation of matrix bone (osteoid) by osteoblast. This process comprises three phases: Activation of resorption activity by osteoclast cells, reversal of bone resorption into deposition of new bone, and bone mineralization by osteoblasts [9]. The process of bone formation and resorption requires tight coordination between the cells. However, disruption of either bone formation or bone resorption could lead to the progression of bone diseases, such as osteoporosis and osteomalacia, hence increasing the risk of bone fracture [10].

**Figure 1:**
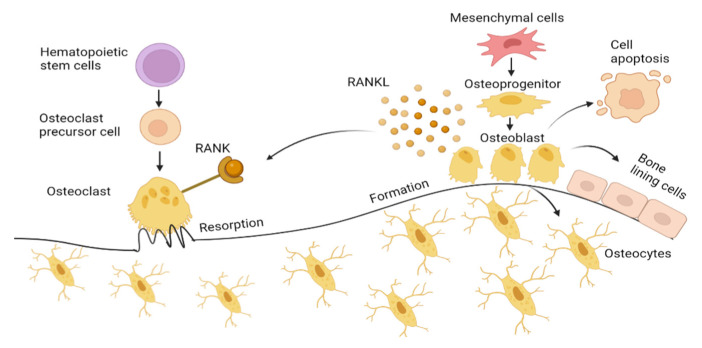
Bone formation by osteoblast and bone resorption by osteoclasts. Mesenchymal cells differentiate into osteoblasts transforming into bone lining cells and osteocytes embedded in mineralized bone matrix. Osteoblast also undergoes apoptosis, which is induced by oxidative stress. Receptor activator of NF-ΚB ligand (RANKL) on osteoblast binds to RANK expressed by osteoclasts, activating osteoclast cells, facilitating osteoclastogenesis by secreting hydrogen ions and lysosome enzyme (Cathepsin K) into the microenvironment under the ruffled border forming resorption pit. Figure generated by biorender.com.

Osteoblasts, originating from mesenchymal stem cells (MSCs), significantly influence bone formation during the remodeling process. MSCs differentiate into osteoprogenitors, also known as pre-osteoblasts, before eventually progress into osteoblasts mediated by Runt-related transcription factor (RUNX2), Distal-less homeobox 5 (Dlx5), and osterix [11]. Osteoblasts synthesize osteocalcin (OCN), alkaline phosphatase (ALP), and collagen type 1 alpha 1 (Col1α1), which accounts for about 90% of the total organic components of bone and is responsible for the bone strength [12,13]. The mature osteoblast undergoes apoptosis, bone lining cells or is incorporated into the mineralized bone tissue as osteocytes [14]. Osteocytes act as mechanosensors that direct the activity of both osteoblasts and osteoclast, as illustrated in Figure 1 [15].

Osteoclasts are derived from hematopoietic stem cells. With the presence of macrophage colony-stimulating factor (M-CSF), osteoclasts precursor cells form a multinucleated osteoclast. Osteoclasts differentiation is mediated by the M-CSF and RANKL, both expressed by osteoblasts. This directly induces osteoclast activation by binding to RANK (receptor) that presents on osteoclasts. This RANKL interaction with the RANK receptor leads to the induction of signaling cascades, which promotes the expression of osteoclastogenesis genes, such as a nuclear factor of activated T-cell 1 (NFATc1), dendritic cell-specific transmembrane protein (DC-STAMP), tartrate-resistant acid phosphatase (TRAP), and cathepsin K, which resorbs the bone. Osteoprotegerin (OPG), which is also secreted by osteoblasts, inhibits osteoclast differentiation by binding to RANKL, blocking RANK–RANKL interaction.

Current treatment and prevention of bone diseases, such as osteoporosis, bone metastases and Paget’s disease, include anti-resorptive and anabolic agents. Anti-resorptive agents mainly comprise bisphosphonates (alendronate, risedronate, ibandronate, and zoledronic acid), calcitonin, denosumab, and selective estrogen receptor modulator (raloxifene) [16,17,18,19]. These treatment options are indeed effective in preventing bone loss by inducing apoptosis of osteoclasts [20]. However, their long-term administration may lead to undesirable side effects, such as gastrointestinal reactions, blood clots, bone fracture, osteonecrosis of jaw, nausea, chest pain, and cardiovascular diseases [21,22,23]. The use of raloxifene has reported several side effects in clinical trials, such as vaginal bleeding, stroke, and breast cancer [24], while the anabolic drug, teriparatide, requires daily administration that can be very costly [25]. Despite the availability of current therapeutic approaches, the treatment cost and side effects associated with these medications have always been a major concern. Hence, there is a need for the development of improved treatments with minimal or no side effects. Most recently, researchers have proposed a safer option, that is to exploit the natural products which offer tremendous health benefits [26].

The marine environment has gained considerable attention in drug research and development of novel drugs for the treatment of many human diseases [27]. Marine organisms are exposed and can adapt to a variety of extreme conditions, such as high salinity, extreme hydrostatic pressure, and temperature. This results in the production of metabolically bioactive compounds, i.e., lipids, pigments, polyphenols, proteins, vitamins, and minerals that possess a wide array of biological activities, such as anticancer, antioxidant, and antimicrobial [28,29,30]. With the growing interest in alternative treatments, marine-isolated compounds can offer benefits in the management of bone diseases as they have demonstrated bone health effects, such as increased bone mineral density, trabecular bone volume, osteoblast mineralization, and inhibitory effect on osteoclastogenic [19,31,32,33]. For example, hymenialdisine, the metabolite of a marine sponge, has been shown to inhibit bone loss in osteoporosis female rat model via suppression of downstream signaling mechanisms leading to osteoclast inactivation [34]. It was also reported that water-soluble matrix isolated from the nacre of marine oyster could upregulate osteoblast differentiation, as shown by increased bone formation markers in osteoblastic cell line [35]. Fucoidan derived from brown algae also showed an anabolic effect through increased bone density and prevented microarchitectural deterioration in an osteoporosis rat model [36].

To date, many marine organisms are being explored for the production of bioactive compounds [30]. Among them is astaxanthin, a naturally occurring red pigment that belongs to the xanthophylls family [37]. Astaxanthin is a great source of natural antioxidants [38] that possess bone-regenerative properties [39]. Despite a rising number of studies proving the potency of astaxanthin in the prevention and treatment of bone diseases, to the best of our knowledge, its clinical application is still scarce. Therefore, this review aims to discuss and provide up-to-date findings on astaxanthin’s beneficial effects on bone health, as well as the evidence from in vitro and in vivo models.

## 2. Astaxanthin

**Figure 2:**
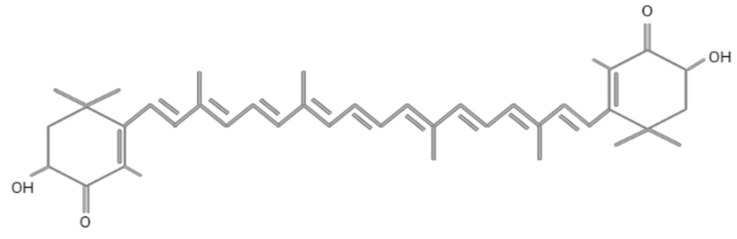
Molecular structure of astaxanthin. Figure generated by biorender.com.

Astaxanthin (3,3′-dihydroxy-β, β′-carotene-4,4′-dione) was first discovered in lobster by Richard Kuhn in 1938 [40]. Today, it is employed in various fields, including food, feed, cosmetics, pharmaceutical, and nutraceutical industries. Astaxanthin has a molar mass of 596.84 g/mol with a molecular formula of C_4_OH_5_2O_4_ and encompasses two terminal β rings joined by a polyene chain, as shown in Figure 2. The presence of hydroxyl (-OH) and carbonyl (C=O) at the carbon third, third (C3,C3′) prime and four (C4) contribute to its unique characteristics, such as the ability to undergo an esterification process, polar nature, and potent antioxidant activity compared to other carotenoids [41]. This compound has the ability to quench singlet oxygen and scavenge free radicals, as reported in numerous studies [42,43], and exhibits ten times higher antioxidant activity as compared to zeaxanthin, lutein, canthaxanthin, and β-carotene, while it is 100 times higher than α-tocopherol [44]. The antioxidant activity of astaxanthin mainly depends on the orientation of hydrogen atoms at the C3 methine and the presence of a double bond, as it donates electrons and reacts with the free radicals to produce a stable product [45,46]. The conjugated double bond comprises a series of carbon-carbon double bonds alternating with carbon-carbon single bonds, located at the compound’s middle segment that determines the pink and red coloration of astaxanthin [43]. 

This antioxidant exists as three different stereoisomers, namely (3S, 3‘S), (3R, 3‘R), and mesomere (3R, 3‘S), depending on the position of two hydroxyl groups on the molecule [47]. The chiral configuration of (3S, 3‘S) is ubiquitously found in nature. Synthetic astaxanthin mainly exists in free form, which is produced from petrochemicals consisting of a mixture of three isomers (3S, 3‘S), (3R, 3‘R), and (3R, 3‘S) in a ratio of 1:2:1 [43]. It is most commonly used in aquaculture as a pigment colorant in fish feeds but its use is prohibited in humans due to safety concerns [48]. Astaxanthin can also be found as trans and cis (E and Z), the geometrical isomers due to the configuration of a double bond [49]. This molecule is unstable in its free state and prone to oxidation. By this, astaxanthin binds to one or two fatty acids to form monoester and diester, making it a more stable molecule [50].

Besides lobster, the main natural sources of astaxanthin are microalgae (*Haematococcus pluvialis* (*H. pluvialis*), *Chlamydomonas nivalis*), bacteria (*Agrobacterium aurantiacum*, *Paracoccus carotinifaciens*), yeasts (*Xanthophyllomyces dendrorhous*, *Phaffia rhodozyma*), and other marine organisms, such as trout, shrimps, krill, and crayfish [43,51,52,53,54]. *H. pluvialis* is a green microalga identified as the richest source of astaxanthin due to its capability to accumulate high amounts of astaxanthin (up to 4% per dry weight) [55]. Astaxanthin synthesis from *H. pluvialis* can be divided into two stages: Green vegetative growth phase (green stage) and reddish inductive production phase (red stage). During the green stage, algae grow in favorable conditions, such as low light and high nitrogen supply. Once algae cells reach high cell density, they are ready to enter the red stage and are exposed to harsh conditions, such as limited nitrogen supply and low light intensity. This stage produces a potent active compound astaxanthin, giving the alga its pinkish-red color [56]. Natural astaxanthin from *H. pluvialis* has been reported to be safe as a dietary supplement with no adverse effects on human health [57]. To date, studies have not claimed any side effects associated with the use of appropriate oral doses of astaxanthin [58,59]. However, high dose intake of astaxanthin could result in pigmentation in animals [60].

By virtue of this, several studies have been performed to demonstrate the benefits of astaxanthin in various aspects of health, including the neuroprotection [61,62,63], cardiovascular [64,65,66], skin [67,68], and for the treatment of various cancers [69]. In addition, emerging evidence has shown that astaxanthin also possesses anti-lipid peroxidation by providing therapeutic effects against atherosclerosis [70] and shows anti-inflammatory properties by inhibiting expression of NF-κB and AP-1 transcription factors as well as inflammatory cytokine production that is responsible for cardiovascular diseases [71]. Apart from that, this compound was reported to possess anti-diabetic properties by lowering the blood glucose level and oxidative stress associated with dysfunction of pancreatic β-cell [72] and anti-cancer properties by suppressing proliferation of human colon cancer cell [73]. Astaxanthin has also been studied for its potential to inhibit bone loss by inducing bone formation and suppressing bone resorption to promote bone health [39,74]. These effects will be described in detail throughout the following section.

## 3. Astaxanthin for Bone Diseases

Free radicals and reactive oxygen species (ROS), such as superoxide anions, hydrogen peroxide, and hydroxide ions, are highly reactive molecules that are constantly formed in our body as a by-product of oxygen molecules in response to aerobic respiration and cellular metabolism [75]. However, if this state of equilibrium is disrupted, the reactive species can overpower the body’s antioxidant defenses, resulting in oxidative stress, which has detrimental effects on lipids, proteins, and deoxyribonucleic acid (DNA) [76]. Oxidative stress is also associated with the pathogenesis of various diseases, including osteoporosis [77].

In bone homeostasis, reactive oxygen species play a dual role: (1) They act as secondary messengers during osteoclastogenesis, regulating the formation of osteoclasts and bone remodeling by breaking down the mineralized matrix, and (2) they inhibit osteoblast activity, leading to increased osteoclast formation and osteocyte apoptosis. Several studies have shown that ROS are involved in osteoclast differentiation through the binding of RANKL-RANK interaction [78,79,80].

During osteogenesis, physiological ROS is generated by activating bone morphogenetic protein-2 (BMP-2) signaling-mediated NADPH oxidase, initiating osteoblast differentiation [81]. However, excessive production of ROS may cause an imbalance of osteoblast and osteoclast activity beyond a threshold that will result in bone loss. It has been demonstrated in both in vitro and in vivo studies that oxidative stress promotes bone loss by inhibiting osteoblast differentiation, osteocyte apoptosis, and bone mineralization, where it positively affects osteoclastogenesis [82,83,84,85,86,87]. Oxidative stress induced by H_2_O_2_ suppressed osteoblastic differentiation of OB-6 osteoblast cells by downregulating osteogenic gene expression (OCN and RUNX2), both important markers required for osteoblast differentiation [88]. Therefore, it is essential to eliminate the accumulated free radicals to prevent their detrimental effects.

Our body has a natural antioxidant defense mechanism to scavenge free radicals, which minimizes the level of ROS [89]. Factors, such as aging, smoking, alcohol consumption, vigorous exercise, UV radiation, and insufficient antioxidant intake, can compromise this defense system [90,91,92,93]. Additionally, suppression of the antioxidant enzymes can result in the accumulation of ROS, which, in turn, can lead to the development of bone diseases and bone loss [94]. Therefore, supplementation of an exogenous antioxidant, astaxanthin, which possesses the ability to effectively counteract oxidative stress, is necessary [95] and may help in disease prevention [95,96,97]. Previously, Chang et al. [74] demonstrated that astaxanthin-loaded liposomes reduced the ROS production induced by lipopolysaccharide (LPS) and decreased the TRAP activity. TRAP is expressed by osteoclast cells and used as a marker for bone resorption activity. It was also noted that astaxanthin-loaded liposomes promoted osteoblast differentiation by increasing ALP activity. Astaxanthin has also been shown in another study to inhibit the expression of osteoclast-specific genes, such as TRAP, Cathepsin K, matrix metalloproteinase (MMP9), and NFATc1 [98]. Astaxanthin also reduced the F-actin formation ring size, indicating osteoclast attachment to the bone and generating the F-actin structure (sealing zone). The sealing ring is a characteristic feature of osteoclast activation for bone resorption [99]. Apart from that, numerous studies have been conducted to demonstrate the beneficial effect of astaxanthin on promoting bone health and provide an alternative approach to hinder the progression of bone diseases, as described in detail below.

### 3.1. Osteoporosis

Osteoporosis is a chronic skeletal disease that can be characterized by a reduction of bone mineral density (BMD), loss of bone mass, degradation of bone microarchitecture, and increased porosity, leading to an increased risk of fracture incidence [5]. This bone disease has been reported as a life-threatening skeletal disease among the elderly, and its prevalence increases due to rapid population aging [100]. The global prevalence of osteoporosis among women and men is 23.1% and 11.7%, respectively [101]. Osteoporosis contributes to the development of fractures commonly occurring at the spine, hip, and wrist, which are associated with mortality, morbidity, and disability [102]. It can significantly impact health and quality of life as patients depend on others for support due to their inability to move.

Osteoporosis is considered asymptomatic until a fracture occurs and it affects more than 200 million people worldwide. According to the World Health Organization (WHO), patients with a T-score lower than 2.5 are considered to have osteoporosis. A T-score is evaluated based on a comparison of an individual’s BMD with the average BMD of normal young population of the same gender [103]. Osteoporosis primarily affects women, especially postmenopausal, due to decreased estrogen hormone levels [104]. Estrogen plays a vital role in bone remodeling by contributing to the formation of bone-forming cells. Other causes of osteoporosis include calcium and vitamin D deficiency, underlying diseases, and glucocorticoid treatments. The risk factors involved in the progression of osteoporosis can be classified as modifiable or non-modifiable. Non-modifiable risk factors include age, gender, and previous family history of osteoporosis, whereas body weight, diet, smoking, and excessive alcohol consumption can be characterized as modifiable risk factors. These modifiable factors cause a significant impact on bone health, contributing to an increase in osteoclast differentiation, bone degradation, and reduced bone mineralization due to excessive oxidative stress.

Many studies have investigated the effects of antioxidants on suppressing oxidative stress that causes osteoporosis [105,106,107]. Previously, the anti-osteoporotic effect of astaxanthin was studied in two separate studies using ovariectomized (OVX)-induced animal model [39,108] (Table 1). OVX female rodents had their ovaries removed to stimulate a condition of estrogen deficiency resembling menopause, which is experienced by postmenopausal women, and they are the most studied animal models that mimic female osteoporosis [4,5]. Both studies showed that astaxanthin exerted positive effects on bone health. The oral supplementation of 10 mg/kg astaxanthin for six weeks increased the BMD of OVX C3H/HeN female mice compared to OVX control (vehicle), estrogen, and 5 mg/kg astaxanthin-treated groups, respectively. Besides, improvement in trabecular formation was reflected through histological analysis, and decreased serum ALP levels were observed in mice treated with astaxanthin, indicating a reduction in bone turnover rate. It was reported that serum calcium and phosphorus levels were also reduced upon administration of astaxanthin, most likely due to the inhibition of skeletal calcium release into the bloodstream [39]. On the contrary, an oral combination of 200 mg/kg SST (soft-shell turtle powder)/OHP (essential oil of *H. pluvialis*), the constituent of astaxanthin, was not able to improve BMD in this animal model but improved bone mineral content (BMC) was seen compared to the sham, OVX control (both treated with 1% carboxymethyl cellulose sodium salt) and 100 mg/kg SST/13 mg/kg OHP groups [108]. Although the authors postulated that the elevation in bone strength and reduction of bone resorption was solely contributed by SST, interestingly, OHP had also shown significant effects on the bone. Thus, a higher dosage of OHP should be used in a future study to exert its predominant impact on osteoporotic bone loss.

Hwang et al. (2018) have also proven the role of astaxanthin in inhibiting osteoclast differentiation in murine bone marrow macrophages (BMMs) treated with 10 ng/mL mouse recombinant receptor activator of nuclear factor-κB ligand (RANKL) and 30 ng/mL macrophage colony-stimulating factor (M-CSF). The cells treated with 30 μM astaxanthin suppressed the expression of osteoclast differentiation-related genes, such as NFATc1, TRAP, DC-STAMP, and cathepsin K, compared to treatment with vehicle (0.1% DMSO). These results also demonstrated that astaxanthin inhibited osteoclastogenesis, as supported by the reduced number of TRAP-positive multinucleated cells [39]. These findings suggested that astaxanthin supplementation improved the osteoporotic manifestation in animal models and had a positive influence on bone loss induced by osteoporosis [39,108].

Enhanced bone anabolic effects as indicated by increased bone structural histomorphometry indices, such as bone volume/total volume (BV/TV), Tb.Th (trabecular thickness), Tb.N (trabecular number) [39,109,110], and BS/TV (bone surface/total volume) [39] after treatment with astaxanthin, were also documented. El Baz and co-authors scrutinized the benefits of astaxanthin in D-gal-induced osteoporosis in male albino rats [109]. In this study, supplementation with 30 mg/kg carotenoid fraction of *H. pluvialis* (CHP) reduced the total serum calcium levels and increased the serum phosphorus levels, an indicator of bone formation. Treatment with CHP also attenuated free radicals and oxidative stress by restoring serum catalase levels, reflecting the ability to counteract oxidative stress owing to its antioxidant properties. CHP reflected more prominent skeletal effects than other treatments due to the carotenoid fraction, which is rich in astaxanthin.

Astaxanthin administration has also been shown to suppress bone resorption by reducing trabecular bone separation (Tb.Sp), osteoclast number (N.OC), osteoclast surface (OC.S), and bone resorption markers: Serum levels of TRAP, TRAP-5b, CTX-1, and cathepsin K [39,108,109,110]. The efficacy of astaxanthin has been examined recently in an irradiation-induced osteoporotic mice model to mimic radiotherapy-induced osteoporosis. Impairment of bone formation and loss due to prolonged radiation exposure during radiotherapy treatment could eventually lead to bone fractures [111,112]. This is manifested through higher production of ROS, elevated activity of osteoclasts, and decreased osteoblast activity, which could further compromise bone health. The oral supplementation of astaxanthin (0.1% astaxanthin mixed in standard mouse diet) was able to reverse the damaging effects of irradiation exposure by improving cortical thickness (Ct.Th), cortical volume (Ct.V), and cortical area (Ct.Ar) when compared to non-supplemented groups [110]. This three-month astaxanthin treatment was shown to exert anti-osteoporotic activity as demonstrated by enhanced osteoblast differentiation marker genes (ALP, bone morphogenetic protein 2 (BMP-2), type I collagen (COL-1), OCN, osterix (OSX), and RUNX-2) [110]. Similarly, several other studies have reported the potential of astaxanthin in bone formation, as indicated by elevated bone formation rate, bone mineralization, osteoblast number, bone formation markers, procollagen type 1 intact N-terminal propeptide (P1NP), and N-terminal middle portion of osteocalcin [39,108,109,110].

**Table 1:** Beneficial effects of astaxanthin on osteoporosis.

Study Model	Interventions	In Vivo	In Vitro	Delivery Mode and Treatment Duration	Significant Findings	References
OVX osteoporotic female C3H/HeN mice *n* = 30 Age: 8 weeks Weight: 21 ± 1	(1) Sham (2) OVX (3) OVX + 17β-estradiol (0.03 µg/head) (4) OVX + AST (5 mg/kg) (5) OVX + AST (10 mg/kg)	✓		Oral and 6 weeks	AST (10 mg/kg) displayed high bone-microarchitecture parameters (BV/TV, BS/TV, Tb.Th, Tb.N) and BMD compared to untreated group.	[39]
BMM cells from femur and tibia of male ICR mice	(1) Control: 0.1% DMSO (2) AST (0.3, 1, 3, 10, 30 µM) Incubated with RANKL (10 ng/mL) and M-CSF (30 ng/mL)		✓	4 days	Suppression of osteoclast differentiation-related genes, such as NFATc1, TRAP, DC-STAMP, and cathepsin K, compared to vehicle.	
OVX osteoporotic female Wistar rats *n* = 56 Age: 8 months Weight: 260–330 g	(1) Sham + water solution of Na-CMC (1%) (2) OVX + water solution of Na-CMC (1%) (3) OVX + 17β-estradiol (0.1 mg/kg) (untreated) (4) OVX + SST1 (100 mg/kg) (5) OVX + SST2 (200 mg/kg) (6) OVX + SST1 (100 mg/kg) + OHP1 (13 mg/kg) (7) OVX + SST2 (200 mg/kg) + OHP2 (26 mg/kg)	✓		Oral and 3 x per week for 6 months	SST2/OHP2 depicted improved bone formation (13.5 ± 0.6 mm) and cortical bone thickness (0.71 ± 0.03 mm) compared to untreated group and SST1/OHP1.	[108]
OST male albino rats *n* = 30 Weight: 130–150 g	(1) Control (2) OST (3) OST + BHP (450 mg/kg) (4) OST + PHP (30 mg/kg) (5) OST + CHP (30 mg/kg)	✓		Oral and 2 weeks	The improvement was observed in OST group treated with CHP compared to untreated groups and other groups.	[109]
Irradiation induced OSTC57BL/6J mice *n* = 30 Weight: 100 g	(1) Control (2) Irradiation induced (3) Irradiation induced + 0.1% of AST	✓		3 months	AST attenuated irradiation induced bone loss by enhancing the bone formation compared to untreated groups.	[110]
Transgenic Aldh2*2 Tg mice Age: 4–12 weeks	(1) 0.1% *w*/*w* of α-tocopherol (2) 0.1% of AST	✓		Oral and 3 months	The BMD of AST-treated group was higher compared to group which received α-tocopherol.	[113]

Abbreviations: OVX, ovariectomized; AST, astaxanthin; SST, soft shell turtle; BHP, biomass of *Haematococcus pluvialis*; PHP, polar region of *Haematococcus pluvialis*; CHP, carotenoid of *Haematococcus pluvialis*; BV/TV, bone volume/total volume; BS/TV, bone surface/total volume; Tb.Th, trabecular thickness; Tb.N, trabecular number; BMD, bone mineral density; Na-CMC, carboxymethyl cellulose sodium salt; OHP, oil of *Haematococcus pluvialis*; Aldh2*2, aldehyde dehydrogenase 2*2; OST, osteoporotic; IR, irradiation; M-CSF, macrophage colony-stimulating factor; NFATc1, nuclear factor of activated T-cell 1; DC-STAMP, dendritic cell-specific transmembrane protein; TRAP, tartrate-resistant acid phosphatase; RANKL, receptor activator of nuclear factor kappa-B ligand.

In a previous study, Hoshi et al. (2020) proved that administration of 0.1% *w*/*w* astaxanthin for three months in an osteoporotic animal model using transgenic Aldh2*2 Tg mice significantly improved BMD compared to rats treated with α-tocopherol, as assessed by dual-energy X-ray absorptiometry (DEXA). In this study, the authors also performed an in vitro experiment to investigate the effects of astaxanthin on the osteoblast differentiation of MC3T3-E1 cells. Murine acetaldehyde-induced osteoblastic MC3T3-E1 cells treated with astaxanthin showed significant upregulation of osteoblastic differentiation markers, such as ALP and Runx2, compared to cells treated with Trolox C, an analog of vitamin E. The ability of astaxanthin to modulate the differentiation of Aldh2*2 Tg murine osteoblastic cells was exhibited by an increment in ALP activity and mineralization. Furthermore, as evaluated by TRAP staining, astaxanthin-treated cells inhibited osteoclast formation. In concordance with the results mentioned above, astaxanthin could be a promising alternative for osteoporosis treatment, and these effects could be mediated by the impact of antioxidant that astaxanthin holds [113].

### 3.2. Osteoarthritis

Disruption in subchondral bone remodeling alters its microstructure and promotes articular cartilage degradation, which is known as osteoarthritis (OA). This chronic degenerative condition predominantly affects hand, hip, and knee joints. In terms of tissue pathology, OA is not only characterized by degeneration of cartilage but also involves changes in the subchondral bone, where the hallmarks of OA include cartilage damage, high subchondral bone turnover, synovial inflammation, and degeneration of ligament and menisci [114]. During early OA, subchondral bone undergoes an altered remodeling process, causing an increase in the expression of RANKL, leading to an elevation of bone resorption by osteoclast leading to sclerosis [115,116,117,118]. Osteoarthritis can be classified as primary, generalized, or localized and secondary, correlated with predisposal factors, such as obesity, previous fracture, and joint disorders [119]. Normal physiological ROS are essential in regulating cartilage homeostasis [120]. The imbalance between ROS production and the inability to neutralize these ROS is a characteristic of OA progression. It leads to an inflammatory response triggering the expression of cartilage degradation markers, MMPs, and chondrocytes apoptosis [121,122]. Cartilage mainly comprises aggrecan, collagen-II (collagen type II), and chondroitin, which is secreted by the chondrocytes.

**Figure 3:**
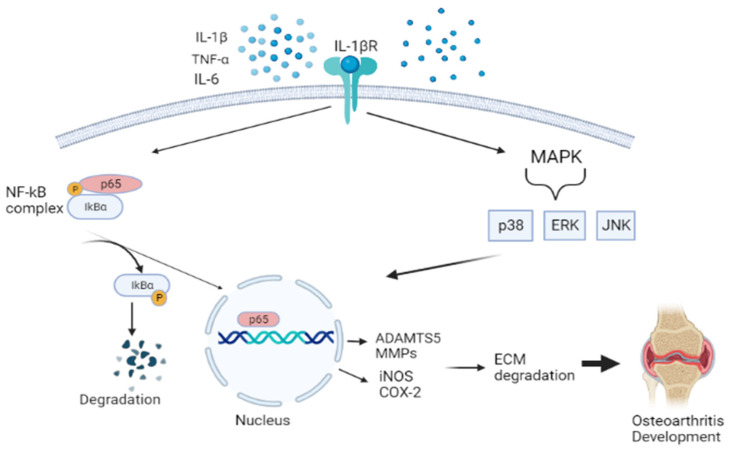
Schematic diagram showing the pathway of nuclear factor kappa beta (NF-Κβ), mitogen-activated protein kinase (MAPK), and c-Jun N-terminal kinases (JNK). This activates aggrecanase and collagenase, leading to ECM degradation and promoting osteoarthritis. Figure generated by biorender.com.

Previous studies have demonstrated that oxidative stress and inflammation significantly contribute to OA pathogenesis [123,124,125,126,127]. During OA, proinflammatory mediators, such as tumor necrosis alpha (TNF-α), interleukin-1β (IL-1β), and IL-6, activate a range of different signaling pathways, including nuclear factor kappa beta (NF-Κβ), mitogen-activated protein kinase (MAPK), and c-Jun N-terminal kinases (JNK) [128]. This promotes cartilage damage and altered cartilage matrix homeostasis, which drives chondrocytes into a catabolic state by producing cartilage-degrading enzymes, matrix metalloproteinases (MMPs) and a disintegrin and metalloproteinase with thrombospondin motifs (ADAMTS), leading to the onset of OA development. NF-Κβ pathway is a transcription factor of many different molecular processes, including inflammation, cell differentiation, and proliferation. This response is initiated by the degradation of the IΚB protein, which is separated from the IΚB-NF-Κβ complex. Phosphorylation of NF- ΚB takes place, translocating to the nucleus. Then results in the inhibition of type II collagen expression and upregulation production of MMPs (i.e., MMP-1, MMP-2, MMP-3, MMP-9, and MMP-13), iNOS, PGE2, NO, and COX-2 [129]. The MAPK pathway, which comprises the extracellular signal-regulated kinases (ERKs), JNK, and p38MAPKs, also aggravates the matrix degradation, as illustrated in Figure 3 [130].

The worldwide prevalence of OA was 300 million in 2017, and it has increased to 500 million since 2019 as reported by the Global Burden of Disease (GBD) [131]. Knee OA is categorized as the 20th highest contributor leading to the disability [132]. The current pharmacological treatment includes non-steroidal anti-inflammatory drugs (NSAIDs), primarily concerned with pain reduction rather than prolonged symptomatic relief [133]. Long-term usage of these drugs may cause side effects, such as kidney failure, gastrointestinal tract complications (i.e., peptic ulcers, perforation, and bleeding), and myocardial infarction [134]. An effective treatment to delay the progression of OA is still scarce. This emphasizes the need to understand OA development and explore a potential alternative treatment to slow down the disease progression in early stages. Researchers are currently focusing on treatment regimens that could target the signaling pathways that initiate OA onset. The osteoarthritic effects of astaxanthin have gained a great interest as a targeted approach in the prevention of OA due to its antioxidant and anti-inflammatory attributes. Astaxanthin possesses cartilage-protective effects, and this has been proven in osteoarthritis models, i.e., destabilization of the medial meniscus (DMM) [135,136], anterior cruciate ligament transection (ACLT) [137], and mono iodoacetate (MIA) [138,139,140] (Table 2).

Nuclear factor-erythroid 2-related factor 2 (Nrf2) activation is an essential pathway in maintaining cartilage structure through its antioxidative defense, which confers protection against OA [141]. As a treatment strategy for OA, Sun and co-researchers aimed to investigate the role of astaxanthin in Nrf2 pathway activation in a DMM OA rat model [135]. The intra-articular injection of astaxanthin twice a week for eight weeks had reduced cartilage degeneration compared to that in the DMM model group which received 10 μL of vehicle solution consisting of 20% Tween-20 (control). This finding was confirmed by an increase in the expression of nuclear factor-erythroid 2-related factor 2 (Nrf2) compared to untreated OA mice groups. This eventually protects the chondrocytes against oxidative damage during OA. The authors also conducted an in vitro study to examine the anti-arthritic effect of astaxanthin. The upregulation of ADAMTS-5, MMP-3, and MMP-13 matrix-degrading enzyme expression was inhibited by astaxanthin. As a result, astaxanthin suppressed cartilage degradation and oxidative stress-induced chondrocyte apoptosis by inactivating the expression of iNOS, COX2, ERK, JNK, p65, and IΚB via the MAPK and NF-Κβ signaling pathways. It increased the expression of collagen type II compared to the untreated chondrocytes. This concludes that astaxanthin could be a promising therapeutic potent antioxidant as a ROS scavenger that can alleviate osteoarthritis caused by oxidative stress [135].

Previous research has acknowledged that chondrocyte ferroptosis is closely linked to cartilage degeneration. Ferroptosis is identified as excessive accumulation of iron, which drives the production of ROS, resulting in orthopedic diseases, including osteoarthritis. Iron is an essential element that plays a vital role in mammalian metabolic processes. Thus, as a potential candidate for preventing OA progression, a recent study by Wang et al. (2020) using a surgically induced rat model of DMM reported that intra-articular injection of astaxanthin (20 mg/kg) twice a week for eight weeks showed smooth, evenly distributed chondrocytes and reduced cartilage abrasion in treated rats compared to that untreated DMM group treated with saline [136]. Astaxanthin suppressed DMM-induced collagen type II reduction, thereby potentiating the ability to protect cartilage from degradation. On top of that, the in vitro experiment on IL-1β-treated rat chondrocytes showed 10 μM astaxanthin could safeguard chondrocytes and delay the development of OA by reducing chondrocyte ferroptosis, which is a significant contributor to the formation and progression of OA.

Another study by Huang et al. (2015) explored the potential protective effect of astaxanthin on cartilage degradation following ACLT surgery in the right knee of rabbits. The authors concluded that intra-articular injection of 50 μM astaxanthin into rabbits once a week for six consecutive weeks ameliorated cartilage loss with reduced lesion severity exacerbated by ACLT. This was accompanied by a significant reduction in MMP-1, MMP-3, and MMP-13 expression, thereby preventing the breakdown of type II collagen and aggrecan (the main components of the extracellular matrix) compared to the DMSO vehicle-treated group [137].

The effect of astaxanthin also has been assessed in the MIA-induced rat model, a well-established model that mimics OA condition in the articular cartilage of humans [142]. Xiong et al. (2022) injected PEG-PTK-PEG@ASTA (0.74 mg/mL) for seven weeks via intra-articular route [139]. The PEG-PTK-PEG@ASTA formulation significantly downregulated the expression of inflammation mediators, such as iNOS and PGE-2. It increased the expression of cartilage genes, such as collagen type II and aggrecan, compared to astaxanthin-treated and unloaded nanoparticle-treated groups. Histological analysis revealed that this formulation decreased the cartilage loss with evenly distributed proteoglycans. In addition, the in vitro findings using BMDM (bone-marrow-derived macrophage) and chondrocytes were consistent with the in vivo study, indicating that PEG-PTK-PEG@ASTA demonstrated superior capabilities compared to other treatment groups. This formulation detected ROS levels and allowed targeted delivery of astaxanthin. In agreement with this study, Çağlar et al. (2021) also found that treatment of 150 μM/mL astaxanthin for four weeks in the same animal model improved the histological scores of normal cartilage and chondrocyte morphology with no osteophyte formation was seen as compared with the HA-treated groups [140]. Similarly, Park et al. (2020) demonstrated that a patented formulation, FlexPro MD^®^ (FP-MD), consisting of natural astaxanthin extracted from *H. pluvialis*, antarctic krill *(Euphausia superba)* oil, and sodium hyaluronate, can protect cartilage, reduce proteoglycan loss, and improve OA pain on rats’ right hind knee following MIA-induction. These results suggested that astaxanthin ameliorated MIA-induced cartilage degeneration [138].

**Table 2:** Beneficial effects of astaxanthin on osteoarthritis.

Study Model	Interventions	In Vivo	In Vitro	Delivery Mode and Treatment Duration	Significant Findings	References
DMM surgery on left knee of male C57BL/6 mice *n* = 24 Age: 8 weeks	(1) Sham group + vehicle: 20% Tween-20 (10 μL) (2) DMM group + vehicle (3) DMM group + 20 mg/kg of AST	✓		Intra-articular injection: 2 x weekly for 2 months	DMM group treated with AST inhibited cartilage degradation and proteoglycan loss compared to untreated groups.	[135]
Chondrocytes from 5 days old C57BL/6J mice	(1) Control (2) OA inducers: 100 μM TBHP, 5 ng/mL TNFα and 5 ng/mL IL-1β (3) 0, 5, 10 or 20 μM AST		✓	2 h	AST downregulated ADAMTS-5, MMP-1,13, collagen type II and iNOS compared to untreated cells.	
DMM surgery on right knee of male Sprague Dawley rats *n* = 32 Age: 8 weeks old	(1) Sham + vehicle: saline (2) DMM + vehicle: saline (3) DMM + Fer-1 (0.5 mg/kg) (4) DMM + AST (20 mg/kg)	✓		Intra-articular injection: 2 x per week for 2 months	AST-treated DMM group showed decreased cartilage abrasion and upregulation of collagen type II compared to untreated groups.	[136]
Chondrocytes derived knee joint cartilage of 7-day old rats	(1) Control (2) IL-1β (10 ng/mL) (3) IL-1β + Fer-1 (1 μM) (4) Fer-1 (1 μM) + AST (10 μM)		✓	24 h	AST inhibited IL-1β induced increase of iNOS, COX2, MMP-13, and delayed OA by modulating GPX4 and SLC7A11 pathway in contrast to the negative control groups (control and IL-1β).	
New Zealand ACLT induction arthritis rabbit *n* = 8 Weight: 2.0–2.5 kg	(1) Sham (control) (2) ACLT group + vehicle: DMSO (0.3 mL) (3) ACLT group + AST (50 μM)	✓		Intra-articular injection: 1 x weekly for 6 weeks	The cartilage loss and expression of MMP-1, 3, 13 were lowered in AST treated compared to vehicle-treated group.	[137]
Right hind knee MIA-induced arthritis male Sprague-Dawley rat *n* = 48 Age: 7 weeks Weight: 200–214 g	(1) Sham + corn oil (5 mL/kg) (2) MIA-induced OA + corn oil (5 mL/kg) (3) MIA-induced OA + Celecoxib (3 mg/kg): Positive control (4) MIA-induced OA + FP-MD (25 mg/kg) (5) MIA-induced OA + FP-MD (50 mg/kg) (6) MIA-induced OA + FP-MD (100 mg/kg) (7) *FP-MD consists of 321 mg of antarctic krill *(Euphausia superba)* oil, 25–35 mg of astaxanthin from *H. pluvialis*, 33 mg of sodium hyaluronate, and remaining 73–83 mg of excipients	✓		Oral gavage daily for 7 days before OA induction and for 21 days post-induction	FP-MD (25, 50, or 100 mg/kg) minimized cartilage damage and OA-associated pain by suppressing proinflammatory cytokines compared to untreated groups.	[138]
MIA-induced Sprague Dawley rats (8 weeks)	(1) PBS (50 μL) (2) AST (0.074 mg/mL) (3) PEG-PPG-PEG (0.74 mg/mL) (4) PEG-PTK-PEG (0.74 mg/mL) (5) PEG-PPG-PEG@AST (0.74 mg/mL) (6) PEG-PTK-PEG@AST (0.74 mg/mL)	✓		Intra-articular injection at OA knee and 7 weeks.	Expression of cartilage markers MMP-2,9,13, IL-1β, TNF-α, PGE-2 was lower and followed by smooth articular surface, high OARSI score in PEG-PTK-PEG@ AST group compared to unloaded astaxanthin group.	[139]
Chondrocytes derived tibia and femur of 5 days old Sprague Dawley rats	(1) Control (2) PBS + 10 ng/mL IL-1β (3) AST (10 μg/mL) + 10 ng/mL IL-1β (4) PEG-PPG-PEG (100 μg/mL) + 10 ng/mL IL-1β (5) PEG-PPG-PEG@ASTA (250 μg/mL) + 10 ng/mL IL-1β (6) PEG-PTK-PEG (500 μg/mL) + 10 ng/mL IL-1β (7) PEG-PTK-PEG@ASTA (750 μg/mL) + 10 ng/mL IL-1β		✓	24 h	PEG-PTK-PEG@ASTA increased expression of collagen type II and aggrecan in the IL-1β-treated chondrocytes compared to nanoparticles without AST.	
Left knee MIA-induced arthritis male Wistar rats	(1) MIA-induced + AST (150 μM/mL) (2) MIA-induced + 2 mg/mL CS (3) MIA-induced + 50 μg/mL HA (4) MIA-induced + AST + HA	✓		Intra-articular injection and 4 weeks	AST-treated group showed greater histological scores compared to CS and HA groups.	[140]
LPS-induced arthritis male C57BL/6 mice *n* = 25 aged 8–10 weeks old weight was not reported	(1) LPS-induced (2) LPS-induced + PBS group (3) LPS-induced + indomethacin (1 mg/kg) (4) LPS-induced + FP-MD (33 mg/kg) (5) LPS-induced + FP-MD (67 mg/kg)	✓		Oral treatment before and after LPS induction at an interval of 2 days for 2 weeks	Mice treated with 33 or 67 mg/kg reduced expression of proinflammatory cytokines and markers associated with OA compared to LPS-induced control group.	[143]
RAW264.7 cells	(1) Control (2) 0 μg/mL (FP-MD) + 100 ng/mL LPS (3) 10 μg/mL (FP-MD) + 100 ng/mL LPS (4) 33 μg/mL (FP-MD) + 100 ng/mL LPS (5) 100 μg/mL (FP-MD) + 100 ng/mL LPS		✓		LPS-FP-MD-treated cells suppressed the expression of inflammatory biomarkers: IL-6, TNF-α, IL-1β compared to cells treated with LPS only.	
Chondrocytes isolated from the OA patient’s cartilage undergoing total knee arthroplasty.	(1) Control (no treatment) (2) 10 ng/mL IL-1β + AST (1 μM) (3) 10 ng/mL IL-1β + AST (10 μM) (4) 10 ng/mL IL-1β + AST (50 μM)		✓	1 h	AST reversed IL-1β-induced activation of ERK ½, p38, and IΚB-α through inhibiting NF-Κβ, MAPK pathways compared to untreated cells.	[144]
SW-1353 human OA chondrosarcoma cells.	(1) control cells (no treatment) (2) 10 ng/mL IL-1β + AST (0 μmol) (3) 10 ng/mL IL-1β + AST (0.01 μmol) (4) 10 ng/mL IL-1β + AST (0.1 μmol) (5) 10 ng/mL IL-1β + AST (1.0 μmol)		✓	48 h	AST suppressed expression of MMP-13, PGE-2, IL-6, TNF-α, which was associated with arthritis compared to untreated cells.	[145]

Abbreviations: DMM, destabilization medial meniscus; ACLT, anterior cruciate ligament transection; MIA, monoiodoacetate; OA, osteoarthritis; DMSO, dimethyl sulfoxide; LPS, liposaccharide; EPA, eicosapentaenoic acid; AST, astaxanthin; CS, corticosteroid; HA, hyaluronic acid; PEG, polyethylene glycol, PTK, poly thioketal; *H. pluvialis*, *Haematococcus pluvialis*; Fer-1, Ferrostatin-1; TNF-α, tumour necrosis alpha; IL-1β, interleukin-1β; IL-6, interleukin-6; NF-Κβ, nuclear factor kappa beta; MAPK, mitogen activated protein kinase; JNK, c-Jun N-terminal kinases; MMPs, matrix metalloproteinases; (ADAMTS), disintegrin and metalloproteinase with thrombospondin motifs; GPX-4, glutathione peroxidase 4; ERK, extracellular signal-regulated kinases; SLC7A11, soluble carrier family 7 Member 11.

There were several studies carried out in the past to scrutinize the effects of astaxanthin on the cartilage degradation markers, such as oligomeric matrix protein (COMP), CTX-11 [138], MMP-1 [143], MMP-2 [138,139,143], MMP-9 [138,139], and MMP-13 [139]. The oral delivery of 25, 50, or 100 mg/kg FP-MD daily for one week before OA induction and three weeks post-induction has been shown to reduce the catabolic factors and proinflammatory mediators of OA, such as TNF-α, IL-1β, IL-6, iNOS, and COX-2, compared to the group treated with 5 mL/kg corn oil [138]. Incongruent with a previous study, Park et al. (2016) reported that supplementation of 33 or 67 mg/kg of FP-MD pre- and post-LPS induction at an interval of two days for two weeks had exhibited increased production of IL-10, an anti-inflammatory cytokine compared with the phosphate buffered saline group (control). This cytokine mediated the chondroprotective effect. Hence, astaxanthin could be proposed as an effective treatment strategy in the early stage of OA. However, this formulation requires further investigation to treat osteoarthritis in clinical settings [143].

Previously, two in vitro studies using human OA chondrosarcoma cells and chondrocytes extracted from the cartilage of OA patients who underwent total knee arthroplasty revealed that astaxanthin possesses anti-arthritic effects [144,145]. Both cell lines were stimulated with 10 ng/mL of IL-1β to mimic inflammatory response, and astaxanthin was found to downregulate the expression of MMP-1, MMP-3 [144], and MMP-13 [144,145], induced by IL-1β in a concentration-dependent manner. This antioxidant was also responsible for reversing the upregulation of p38 (protein38), IkB-α, ERK ½ phosphorylation [144], and catabolic factors: Prostaglandin (PGE2), IL-6, and TNF-α [145], compared to untreated cells, which cause inactivation of NF-Κβ [144,145] and MAPK signaling pathways [145]. This was evidenced by an increase in the antioxidant enzymes SOD and GPX, thereby indicating their antioxidant and anti-inflammatory properties in the development of OA.

In a recent randomized human clinical study, Stonehouse et al. (2022) evaluated the effects of supplementation of krill oil containing astaxanthin for six months on the knee pain score, stiffness, and physical function in mild to moderate knee OA patients (*n* = 235, age = 55.9 ± 6.8 years). This study reported that participants who consumed krill oil experienced improvement in knee pain (krill oil = 17.8% vs. placebo = 12.6%), stiffness (krill oil = 19.5% vs. placebo = 13.1%), and physical function (krill oil = 14.8% vs. placebo = 10.1%) compared to the placebo (each capsule: 1 g/d mixed vegetable oil). These data revealed that krill oil effectively improves pain, stiffness, and physical function in mild-to-moderate knee OA patients [146].

### 3.3. Osteonecrosis

Osteonecrosis (ON) is defined as an orthopedic disease caused by disruption of blood supply to the bone and altered mesenchymal differentiation, resulting in bone cell death [95]. ON can be classified into two major types, namely traumatic (i.e., fracture and joint dislocation) and non-traumatic, due to adverse effects of long-term use of medication, such as corticosteroid [147], chemotherapy treatment for cancer patients, and excessive alcohol consumption [148]. It is usually associated with impaired subchondral microcirculation, predominantly in the primary blood supply to the femoral head and retinacular vessel. This results in the development of microfractures in the necrotic site due to compromised bone remodeling and could be linked to a substantial risk of secondary osteoarthritis.

The most commonly affected areas for ON are the hip joint, also known as the femoral head, knee, and humeral head. ON ranks as the third most common cause of total hip arthroplasty among patients under 50 in the United Kingdom (UK) [149]. It was postulated that oxidative stress plays a crucial role in the pathogenesis of ON where supplementation of an antioxidant had been shown to reduce oxidative damage in a steroid-induced rabbit model [150,151].

The current pharmacological treatment for ON includes anticoagulants, bisphosphonates, statins, and vasodilator drugs [152,153,154]. However, the benefits of these treatments are primarily in the early stages. There are no clear recommendations for their usage in patients with ON [155,156]. Thus, this necessitates the development of an effective treatment for a long-term management of ON [154]. As an alternative, astaxanthin supplementation was suggested to overcome the progression of ON. There was only one study reported on the effects of astaxanthin and osteonecrosis (Table 3) (i). In a study by Wiradiputra et al. (2018), it was demonstrated that astaxanthin had suppressed bone cell death in alcohol-induced osteonecrosis male rat model [157]. It was well-documented that excessive alcoholism may contribute to a higher risk of femoral head osteonecrosis, where it suppresses bone mesenchymal stem cells (BMSCs) and osteoblast differentiation, leading to altered regeneration of bone [158]. The authors reported that the number of osteocytes and osteoblasts from the trabeculae of the femoral head increased significantly in the alcohol-induced mice treated with astaxanthin (0.106 mg/kg/day) via intraoral for three weeks compared to the control group. This finding suggested that using astaxanthin as a potent antioxidant could contribute to osteogenesis and osteoblast formation. However, as only one study has explored the potential use of astaxanthin in alleviating osteonecrosis [157], further investigation is warranted to prove the efficacy of astaxanthin in osteonecrosis treatment.

### 3.4. Osteosarcoma

Osteosarcoma (OS) is a skeletal disease characterized by malignant bone cancer, and the prevalence of this disease is significantly higher during adolescence (15–21 years) and in the elderly (75–79 years). The prevalence is associated with specific ages due to rapid growth spurt and an increased bone resorption [159]. It primarily affects the metaphyseal region of bone, proximal tibia, distal femur, proximal humerus, and distal radius [160].

OS was reported to impact about 3.4 per million people annually worldwide [161], approximately one to three cases per million [162,163]. Bone sarcomas, originating from MSC, are characterized by malignant osteoblast, which produces an osteoid matrix. Moreover, uncontrolled oncogene expression due to mutation, high bone turnover, and aberrant activation of wingless signaling (Wnt) creates the condition for tumor progression [164,165]. Most OS patients tend to develop lung metastases associated with reduced survival rates compared to those without metastases [166]. The presence of swelling and pain primarily identifies OS and, in severe cases, could lead to bone fracture [167]. The current treatment options include surgery, chemotherapy, and immunotherapy, while the neoadjuvant chemotherapy options are methotrexate, doxorubicin, and cisplatin [168,169]. The application of chemotherapy drugs decreases tumor size and reduces the recurrence rate [170]. However, there is a high possibility that healthy cells will be attacked due to the unspecific mode of action of these chemotherapeutic drugs. The adverse effects of long-term use of these medications could compromise the patients’ quality of life. Furthermore, toxic effects, such as renal failure, cardiotoxicity, liver and nerve damage, gastrointestinal disturbances, and survival rates of only 20%, hinder the efficacy of the treatment [133,171].

Previously, astaxanthin supplementation was suggested and utilized in in vitro studies using cell lines to evaluate the effect of this antioxidant on osteosarcoma [172,173] (Table 3) (ii and iii). The study demonstrated that astaxanthin (5, 10, or 2.5 μM) significantly reduced the proliferation of three canine osteosarcoma cell lines (canine osteosarcoma cell lines: OS 2.4, HMPOS, and D17) compared to untreated cell [172]. This markedly suggested the antiproliferative effects of astaxanthin in inhibiting cancer cell proliferation and inducing apoptosis with chemopreventive activity. Astaxanthin was also noted to suppress osteosarcoma cell proliferation by increasing the expression of osteogenic markers (Runx2, collagen type 1, osteopontin, and osteocalcin) in MG63 cells compared to untreated cells [173]. Thus, these studies suggested that astaxanthin is beneficial in application for bone cancer treatments. However, additional in vitro and in vivo investigations on using astaxanthin as an antiproliferative agent are necessary.

**Table 3:** Beneficial effects of astaxanthin on osteonecrosis and osteosarcoma.

Study Model	Interventions	In Vivo	In Vitro	Delivery Mode and Treatment Duration	Significant Findings	References
Alcohol-induced ON of femoral head of male Wistar rats *n* = 24 Age: 10–14 weeks Weight: 200–250 g	(1) Control: 0.5 ml of 40% alcohol (2) 0.106 mg/kg/day of AST	✓		Intraoral for 3 weeks	AST significantly increased number of osteocytes and osteoblasts compared to control group.	[157]
MG-63 human OS cell line	(1) Control: DMSO (2) AST (10, 20, and 40 µM)		✓	24 h	AST significantly increased expression of Runx2, Col-type 1, OPN, and OCN compared to control group.	[173]
Canine OS cell lines: OS 2.4, HMPOS and D17	Cell growth Experiment: (1) Culture medium (Control) (2) THF (vehicle) (3) AST diluted in TEF (1.25, 2.5, 5, and 10 µM)		✓	8 days	Sarcoma cell lines treated with AST inhibited cell proliferation compared with control and vehicle.	[172]
	Antioxidant potential: (1) THF (vehicle) (2) AST (10 µM) (3) THF + HP (200 µM) (4) AST + HP (200 µM)		✓	12 h	Antioxidant potential of cell treated with AST significantly increased compared with vehicle.	

Abbreviations: ON, osteonecrosis; Col-type 1, collagen-type 1; OPN, osteopontin; OCN, osteocalcin; OS, osteosarcoma; THF, tetrahydrofuran; HP, hydrogen peroxide; RUNX-2, Runt-related transcription.

## 4. Limitations and Future Directions

In this review, astaxanthin has been studied in numerous bone disease models and has shown promising results as a potent antioxidant. The protective effects of astaxanthin in enhancing bone strength, bone mineral density, and osteoblast differentiation, inhibiting osteoclast activation and cartilage degradation, have been proven. Therefore, astaxanthin would be a promising candidate for an alternative treatment of oxidative stress-related bone diseases. However, the current data from the studies that we reviewed only focus on the effect of astaxanthin on a few bone-related pathologies. The potential impact of astaxanthin should also be elucidated on other oxidative stress-related bone disorders, such as bone fracture, osteomalacia, osteomyelitis, and Paget’s disease. Secondly, only one human clinical study has highlighted the positive effect of astaxanthin in knee OA, thus far. More studies are needed, particularly in human clinical studies, to support the pre-clinical data gathered. Despite astaxanthin’s promising outcomes in bone health, its applications as a pharmaceutical product could be hindered due to low bioavailability [174]. Apart from that, rigid bone structure also influences the amount of drug available in the bone [175]. In the near future, it is suggested that drug delivery systems should be exploited to overcome these drawbacks by encapsulating astaxanthin into a suitable vehicle for enhanced bioavailability, stability, and sustained release of astaxanthin [176,177,178,179,180].

Furthermore, the dosage of astaxanthin should be optimized in future clinical studies to demonstrate the improvement and efficacy which can be recommended to patients according to their requirements. Moreover, an optimized dose of astaxanthin is necessary to ensure the desired therapeutic effect is achieved while the risk of systemic adverse effects is minimized. This can lay an essential basis for future studies on interpreting the clinical trial results to ensure patient safety and therapeutic benefits. On top of that, in a few experimental studies, astaxanthin was also formulated along with other active ingredients, which might compromise its predominant effects. Thus, the potency of pure astaxanthin compound should be explored in future bone-related studies.

## 5. Conclusions

In summary, the findings in this review have demonstrated that astaxanthin, a well-known antioxidant, had a positive effect on common bone diseases, including osteoporosis, osteoarthritis, osteosarcoma, and osteonecrosis. Different astaxanthin dosages and formulations, and combination with other compounds, have been reviewed. The bone protective effects of astaxanthin are attributed to their ability to promote bone mineralization, increased osteoblast differentiation, bone microarchitecture, and reduced osteoclast formation through scavenging excess free radicals, which is a net product of oxidative stress.
